# Administration of an LXR agonist promotes atherosclerotic lesion remodelling in murine inflammatory arthritis

**DOI:** 10.1002/cti2.1446

**Published:** 2023-04-18

**Authors:** Dragana Dragoljevic, Man Kit Sam Lee, Gerard Pernes, Pooranee K Morgan, Cynthia Louis, Waled Shihata, Kevin Huynh, Arina A Kochetkova, Patrick W Bell, Natalie A Mellett, Peter J Meikle, Graeme I Lancaster, Michael J Kraakman, Prabhakara R Nagareddy, Beatriz Y Hanaoka, Ian P Wicks, Andrew J Murphy

**Affiliations:** ^1^ Division of Immunometabolism Baker Heart and Diabetes Institute Melbourne VIC Australia; ^2^ Inflammation Division Walter and Eliza Hall Institute of Medical Research Parkville VIC Australia; ^3^ Rheumatology Unit Royal Melbourne Hospital Melbourne VIC Australia; ^4^ Department of Immunology Monash University Melbourne VIC Australia; ^5^ Department of Surgery Ohio State University Wexner Medical Center Columbus OH USA

**Keywords:** atherosclerosis, cholesterol metabolism, inflammation, monocytes, rheumatoid arthritis

## Abstract

**Objectives:**

The leading cause of mortality in patients with rheumatoid arthritis is atherosclerotic cardiovascular disease (CVD). We have shown that murine arthritis impairs atherosclerotic lesion regression, because of cellular cholesterol efflux defects in haematopoietic stem and progenitor cells (HSPCs), causing monocytosis and impaired atherosclerotic regression. Therefore, we hypothesised that improving cholesterol efflux using a Liver X Receptor (LXR) agonist would improve cholesterol efflux and improve atherosclerotic lesion regression in arthritis.

**Methods:**

*Ldlr* ^*−/−*^ mice were fed a western‐type diet for 14 weeks to initiate atherogenesis, then switched to a chow diet to induce lesion regression and divided into three groups; (1) control, (2) K/BxN serum transfer inflammatory arthritis (K/BxN) or (3) K/BxN arthritis and LXR agonist T0901317 daily for 2 weeks.

**Results:**

LXR activation during murine inflammatory arthritis completely restored atherosclerotic lesion regression in arthritic mice, evidenced by reduced lesion size, macrophage abundance and lipid content. Mechanistically, serum from arthritic mice promoted foam cell formation, demonstrated by increased cellular lipid accumulation in macrophages and paralleled by a reduction in mRNA of the cholesterol efflux transporters *Abca1*, *Abcg1* and *Apoe*. T0901317 reduced lipid loading and increased *Abca1* and *Abcg1* expression in macrophages exposed to arthritic serum and increased ABCA1 levels in atherosclerotic lesions of arthritic mice. Moreover, arthritic clinical score was also attenuated with T0901317.

**Conclusion:**

Taken together, we show that the LXR agonist T0901317 rescues impaired atherosclerotic lesion regression in murine arthritis because of enhanced cholesterol efflux transporter expression and reduced foam cell development in atherosclerotic lesions.

## Graphical abstract

In this study, we revealed that administration of a liver X Receptor (LXR) agonist (T0901317) to atherosclerotic‐prone mice with inflammatory arthritis reduces atherosclerosis and systemic cytokines along with joint inflammation. We have previously shown that arthritic mice have enhanced myelopoiesis because of defective cholesterol metabolism in haematopoietic stem and progenitor cells (HSPCs). However, LXR activation was unable to restore the expression of its target cholesterol efflux genes Abca1, Abcg1 or Apoe in HSPCs and had no impact on myelopoiesis. Instead, we observed an increase in ABCA1 expression in plaque macrophages and reduced foam cell formation along with lower levels of plaque tumor necrosis factor levels.

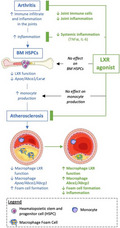

## Introduction

Rheumatoid arthritis (RA) is an autoimmune disease that is associated with increased cardiovascular (CV) events including myocardial infarction (MI) and stroke, and premature mortality.^1^, ^2^, ^3^ Traditional CV risk factors do not explain the increased incidence of CV disease (CVD) in RA, which is instead attributed to systemic inflammation.^4^, ^5^, ^6^, ^7^ Patients with RA have hallmarks of systemic inflammation demonstrated by elevated levels of pro‐inflammatory cytokines, increased C‐reactive protein (CRP),^5^ endothelial dysfunction,^8^ increased carotid artery intimal‐medial thickness and elevated circulating monocytes (monocytosis).^7^ It is well established that monocyte levels are causal to CVD severity, events and mortality.^9^, ^10^, ^11^, ^12^, ^13^

We have recently shown that systemic inflammation in an experimental model of RA causes cellular cholesterol defects in bone marrow (BM) haematopoietic stem and progenitor cells (HSPCs) and monocytosis‐driven atherosclerosis.^14^ Cellular cholesterol accumulation was caused by a reduction in the cholesterol efflux genes ATP binding cassette transporters (*Abc)a1*, *Abcg1* and apolipoproteine (*Apoe*) in BM HSPCs. Mice with genetic deficiencies in these cholesterol efflux genes have pronounced monocytosis and systemic inflammation.^15^, ^16^, ^17^, ^18^, ^19^, ^20^ In our murine model of RA, monocytosis was associated with accelerated atherosclerosis and impaired lesion regression.^14^ Atherosclerotic lesions in the arthritic mice were larger and exhibited an unstable phenotype as evidence by increased immune cell infiltrate and lipid content. Importantly, in murine arthritis and genetic deficiency models of cholesterol efflux, reconstituted high‐density lipoprotein (rHDL) was able to reverse monocytosis and monocyte lesion entry, suggesting that cholesterol metabolism defects may provide an alternative mechanism to explain the increased CVD in patients with inflammatory disorders such as RA.

Cholesterol metabolism is also altered in immune cells in RA. Plasma from RA patients has been shown to downregulate cholesterol efflux proteins resulting in lipid accumulation in a human macrophage cell line.^21^, ^22^ Moreover, HDL functionality is perturbed in patients with active disease and high systemic inflammation.^22^, ^23^ Patients with RA showed an impairment of cholesterol efflux capacity via *ABCA1* and *ABCG1*,^22^ suggesting a defect in cellular cholesterol efflux, resulting in lipid accumulation in macrophages. Impaired macrophage cholesterol efflux favors cellular lipid retention and hence foam cell formation, contributing to the inflammatory milieu of atherosclerotic lesions by not only increasing plaque cholesterol but also increasing inflammatory cytokine and chemokine secretion.

Promoting the expression of cholesterol efflux transporters reduces cellular cholesterol accumulation and ultimately results in reduced atherosclerosis.^24^, ^25^ These cellular cholesterol transporters are transcriptionally regulated by the Liver X Receptor (LXR). LXRs bind to LXR response elements to induce transcription of genes including *Abca1* and *Abcg1*. The antiatherosclerotic effects of LXR activation (using the agonists T0901317 or GW3965) have been well documented and they are considered the ‘master regulators’ of cellular cholesterol efflux, reversing cholesterol transport and also inhibiting inflammation.^26^ A transgenic LXRα mouse model on a low‐density lipoprotein receptor‐deficient (*Ldlr* ^*−/−*^) background, which overexpresses the cholesterol efflux transporters, had an ~80% reduced atherosclerotic lesion size compared with its nontransgenic *Ldlr* ^*−/−*^ counterpart.^27^ Using a pharmacological approach, administration of a synthetic LXR agonist (GW3965) reduced atherosclerotic lesions in *Apoe* ^*−/−*^ mice.^28^ LXR activation can increase cholesterol efflux from the macrophages within the atherosclerotic lesions, promote macrophage egress, and in turn regression of atherosclerotic plaque.^29^ It can also induce cholesterol efflux gene expression in HSPCs and decreases monocyte production.^15^

We hypothesised that an LXR agonist would (1) restore cholesterol efflux genes in BM HSPCs to reduce monocytosis in inflammatory arthritis, (2) promote cholesterol efflux from lesion macrophages, (3) enhance atherosclerotic lesion regression in inflammatory and (4) may have anti‐inflammatory effects on arthritis.

## Results

### Arthritic mice administered the LXR agonist had increased atherosclerotic regression and improved plaque stability

**Figure 1:**
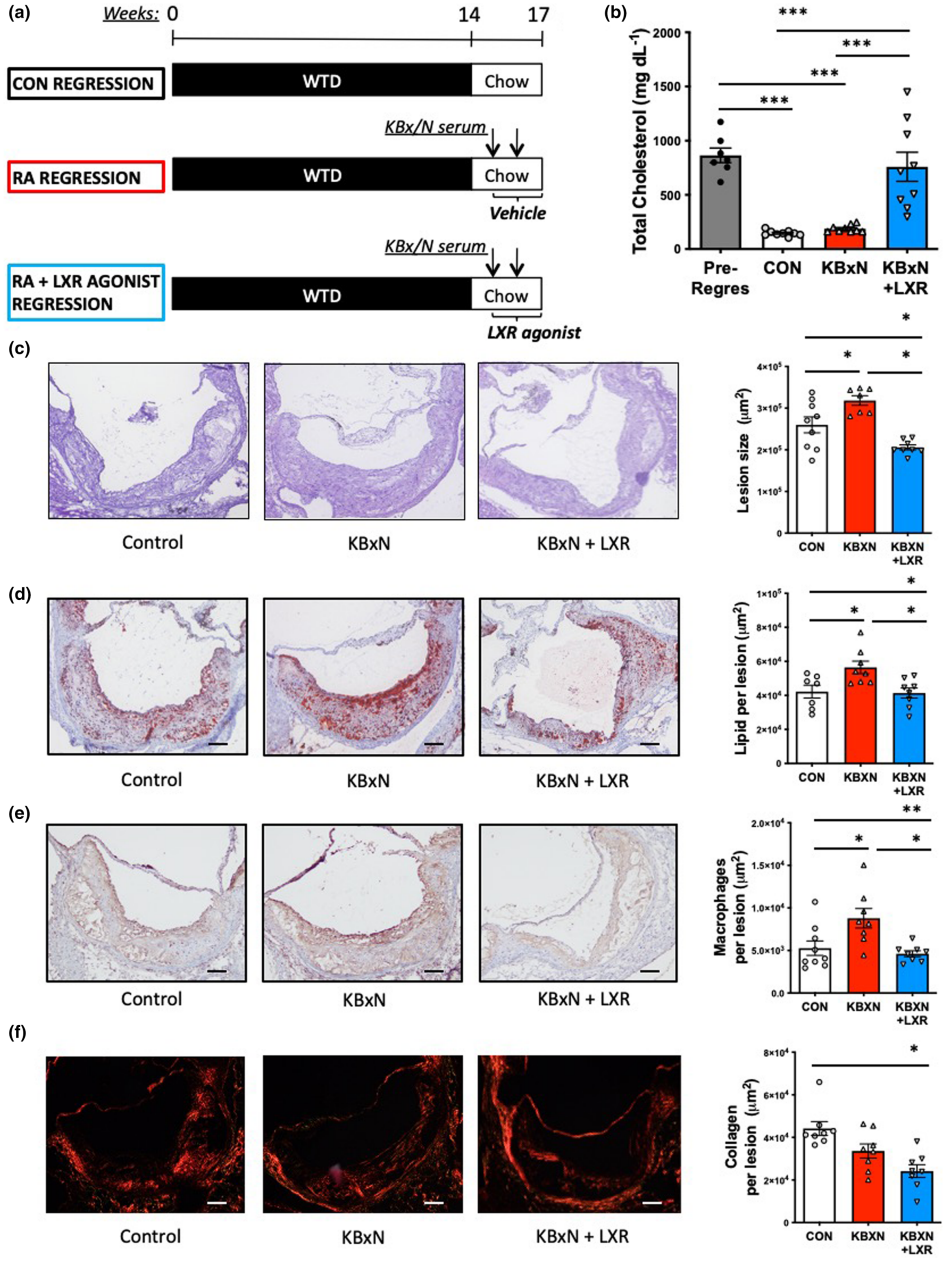
Administration of the LXR agonist improves atherosclerotic lesion regression. **(a)** *Ldlr* ^*−/−*^ mice were fed a western‐type diet (WTD) for 14 weeks to induce atherogenesis then switched to a chow diet to induce lesion regression. Mice undergoing lesion regression were divided into three groups: (1) control, (2) K/BxN serum transfer arthritis (K/BxN) (2× 100 μL K/BxN serum, I.P.) or (3) rendered arthritic and were administered LXR agonist T0901317 (25 mg kg^−1^, I.P., daily) for 2 weeks. **(b)** Total serum cholesterol levels. *n* = 7–9 mice/group. The aortic sinus of *Ldlr* ^*−/−*^ mice was characterised for **(c)** atherosclerotic lesion size (H&E staining), **(d)** lipid abundance (ORO staining), **(e)** macrophage content (CD68^+^) and **(f)** collagen content. Scale bars = 50 μm. *n* = 7–9 mice, in 1 cohort. *P < 0.05, **P < 0.01 as indicated. All data are mean ± SEM.

Patients with RA are generally older individuals with existing atherosclerotic CVD. We have previously shown in murine models and clinical samples of RA, haematopoietic cells have a reduction in the expression of *Abca1* and *Abcg1*.^14^ Given these genes are regulated by the LXR, we were interested in exploring whether LXR activation could reduce already established atherosclerotic lesions. Hence, we employed an atherosclerotic regression model to investigate this. Female 8–10‐week‐old low‐density lipoprotein receptor‐deficient (*Ldlr* ^*−/−*^) mice were fed a western‐type diet (WTD; 0.15% cholesterol) for 14 weeks to initiate atherogenesis. Mice were subsequently switched to a chow diet to reduce circulating cholesterol levels and initiate lesion regression. Following 1 week on a chow diet, mice were either left as controls for regression, rendered arthritic by injecting K/BxN serum,^30^ or rendered arthritic and administered the LXR agonist T0901317 (25 mg kg^−1^, I.P.) daily for 2 weeks (Figure 1a). Three weeks on a chow diet resulted in a substantial reduction in plasma cholesterol in both the control and arthritic mice compared with the pre‐regression levels. Conversely, arthritic mice receiving T0901317 had markedly increased circulating cholesterol levels compared with the other regression groups (Figure 1b), which were comparable to the pre‐regression levels. This is likely because of the well‐known mechanism by which LXR activation in the liver induces lipogenesis and consequently causes hypercholesterolaemia.^31^, ^32^

We also examined the aortic sinus, which revealed impaired lesion regression in arthritic mice compared with controls, demonstrated by increased lesion size (Figure 1c). Remarkably, atherosclerotic lesions of arthritic mice receiving the LXR agonist had smaller lesions than the arthritic mice receiving vehicle, and the nonarthritic, control lesion regression mice (Figure 1d). Having observed dramatically improved lesion regression with LXR agonist treatment in arthritic mice, we further investigated the abundance of plaque macrophages, which are responsible for the majority of cholesterol accumulation, as well as lesional lipid content, as indicators of immune cell infiltration and plaque vulnerability. As we have previously reported,^14^ arthritic mice displayed compromised lesion remodelling, with markedly increased lipid and macrophage content compared with control mice undergoing lesional regression (Figure 1d and e). T0901317 treatment completely restored lipid and macrophage content to that of control regression levels (Figure 1d and e). We next examined the collagen content of the plaques to assess remodelling towards a stable phenotype. We observed a trend for less collagen in the arthritic mice compared with controls, but there was no difference in plaque collagen content between vehicle‐ and T0901317‐treated arthritic mice (Figure 1f). Given that the LXR agonist increased plasma cholesterol, the improved atherosclerotic outcome with T0901317 is likely through an alternative mechanism.

### Arthritis‐driven monocytosis persists after LXR agonist treatment

**Figure 2:**
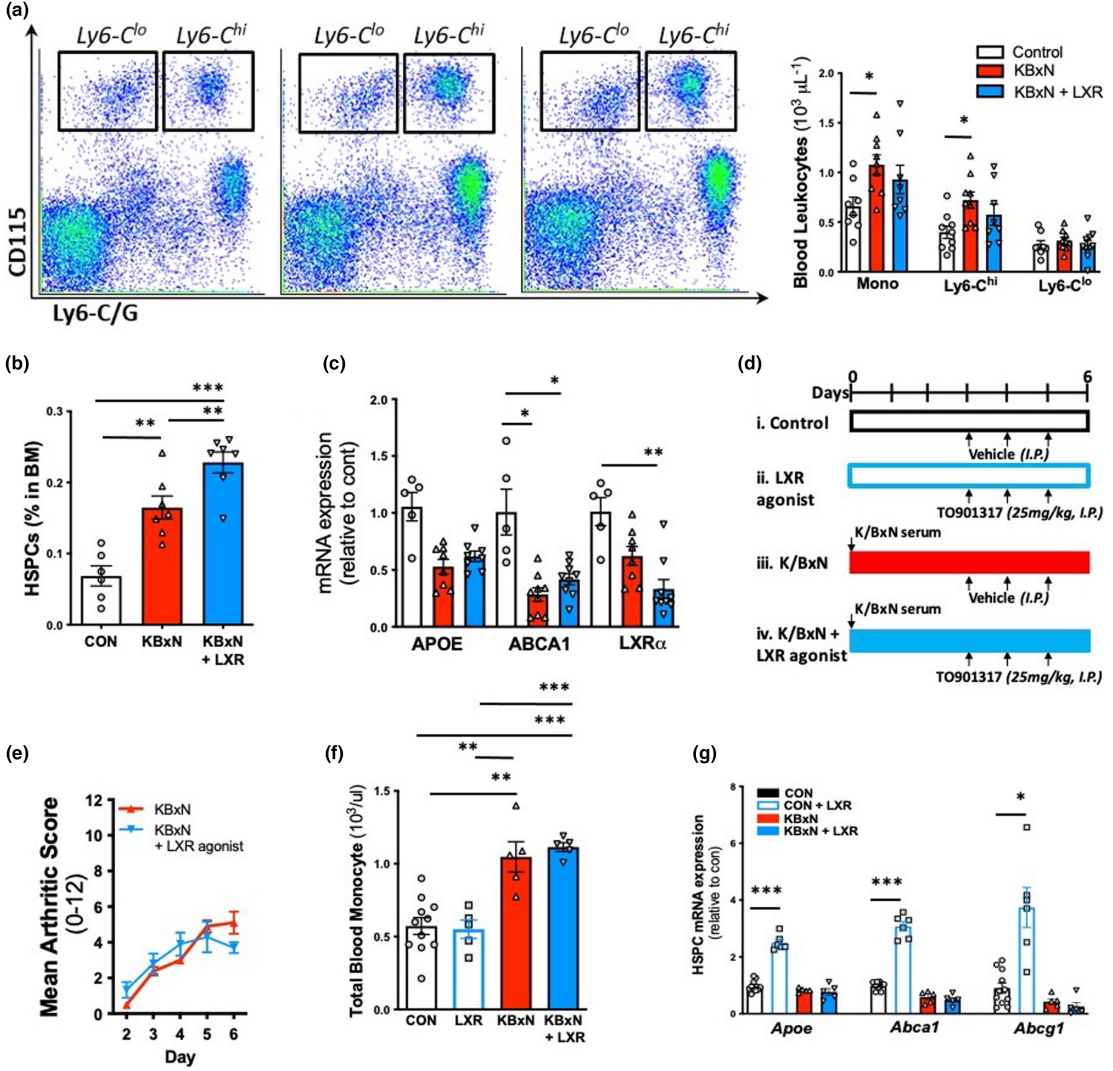
LXR agonist does not attenuate inflammatory arthritis‐driven monocytosis. **(a–c)** From the cohort in Figure 1, blood monocytes were quantified by flow cytometry; **(a)** Representative gating flow plots and quantification. **(b)** BM HSPCs (*n* = 8–10 mice, 1 cohort) and **(c)** gene expression from stem cell‐enriched bone marrow (SCenBM) (*n* = 5–9). **(d–g)** C57Bl/6 Control and K/BxN arthritic mice **(d)** were administered either vehicle or LXR agonist for 3 days (T0901317, 25 mg kg^−1^, I.P., daily), **(e)** clinical score determined, **(f)** blood monocyte abundance assessed by flow cytometry and **(g)** gene expression from HSPCs isolated by FACS. *n* = 5–10, run in 2 cohorts. *P < 0.05, **P < 0.01, ***P < 0.001 as indicated. All data are mean ± SEM.

Lesional macrophages arise predominantly from blood monocytes, which are known to play a vital role in impairing atherosclerotic plaque regression.^33^, ^34^, ^35^ We have previously reported that defects in cellular cholesterol efflux promote enhanced myelopoiesis and ultimately monocytosis, which was associated with impaired lesion regression in RA.^14^ Therefore, we hypothesised that treatment with an LXR agonist will restore cholesterol efflux in BM HSPCs to reduce monocyte production and hence reduce leukocytosis to ultimately improve lesion regression in RA. However, while prominent monocytosis was observed in the arthritic mice compared with control, T0901317 administration did not blunt monocyte production in *Ldlr* ^*−/−*^ mice (Figure 2a). To assess how the LXR agonist is affecting haematopoiesis, we next examined BM stem cells. While BM HSPCs were increased in the setting of arthritis, mice treated with the LXR agonist further expanded the HSPC population (Figure 2b). Furthermore, it is known that hypercholesterinemia can drive BM myelopoiesis,^36^ which may play a role in increased BM HSPCs in this setting, as T0901317 induced marked hypercholesterolaemia. However, the LXR agonist did not reduce myelopoiesis or monocytosis in RA, suggesting lesion regression in the LXR‐treated mice is not through reduction of monocytes.

### BM HSPCs from arthritic mice are not responsive to the LXR agonist T0901317

To understand why LXR activation failed to restore myelopoiesis in RA, we first sought to identify whether the BM stem cells and progenitor cells were responding to LXR activation. Therefore, we enriched the BM for stem and progenitor cells and assessed gene expression of *Lxrα*, as well as its target genes, *Abca1* and *Apoe*. This analysis revealed that 2 weeks of T0901317 treatment failed to upregulate *Lxrα*, *Abca1* and *Apoe*, suggesting that the stem and progenitor cells of arthritic mice were not responsive to the LXR agonist (Figure 2c). Subsequently, we aimed to clarify whether this defect was occurring in the HSPCs which are upstream from both the progenitors and monocytes.

In a separate experiment, we subjected both K/BxN‐induced arthritic and nonarthritic WT mice with either T0901317 treatment or vehicle treatment for 3 consecutive days after the clinical onset of arthritis (Figure 2d and e). Similar to our observations in the *Ldlr* ^*−/−*^ mice, the LXR agonist did not attenuate arthritis‐driven monocytosis (Figure 2f). Monocyte levels in control mice were also unaltered (Figure 2f), as expected (because control mice do not display defects in cholesterol handling). Next, we sought to explore whether T0901317 increases cholesterol efflux gene expression in HSPCs, in both control mice as well as arthritic mice. To do this, we isolated HSPCs using fluorescence‐activated cell sorting (FACS) and performed mRNA expression analysis. As anticipated, 3 days of T0901317 administration induced up to a fourfold increase in *Apoe*, *Abca1* and *Abcg1* gene expression in HSPCs isolate from nonarthritic control mice (Figure 2g). However, similar to data from the stem cell‐enriched BM of *Ldlr* ^*−/−*^ mice from the atherosclerotic regression study, T0901317 was unable to upregulate LXR target genes in HSPCs of arthritic mice (Figure 2g). Taken together, these data reveal that HSPCs of arthritic mice are unresponsive to LXR activation by T0901317.

### LXR agonism reduces plaque foam cell formation in BMDMs

Given that the LXR agonist was able to promote atherosclerotic lesion regression, independent to circulating cholesterol levels and without altering monocyte levels, we explored how T0901317 might be affecting macrophage biology to promote lesion regression in arthritis. Indeed, it is well known that LXR activation in atherosclerotic lesions can induce macrophage cholesterol efflux, reduce cellular lipid accumulation and promote macrophage egress from plaque.^29^

**Figure 3:**
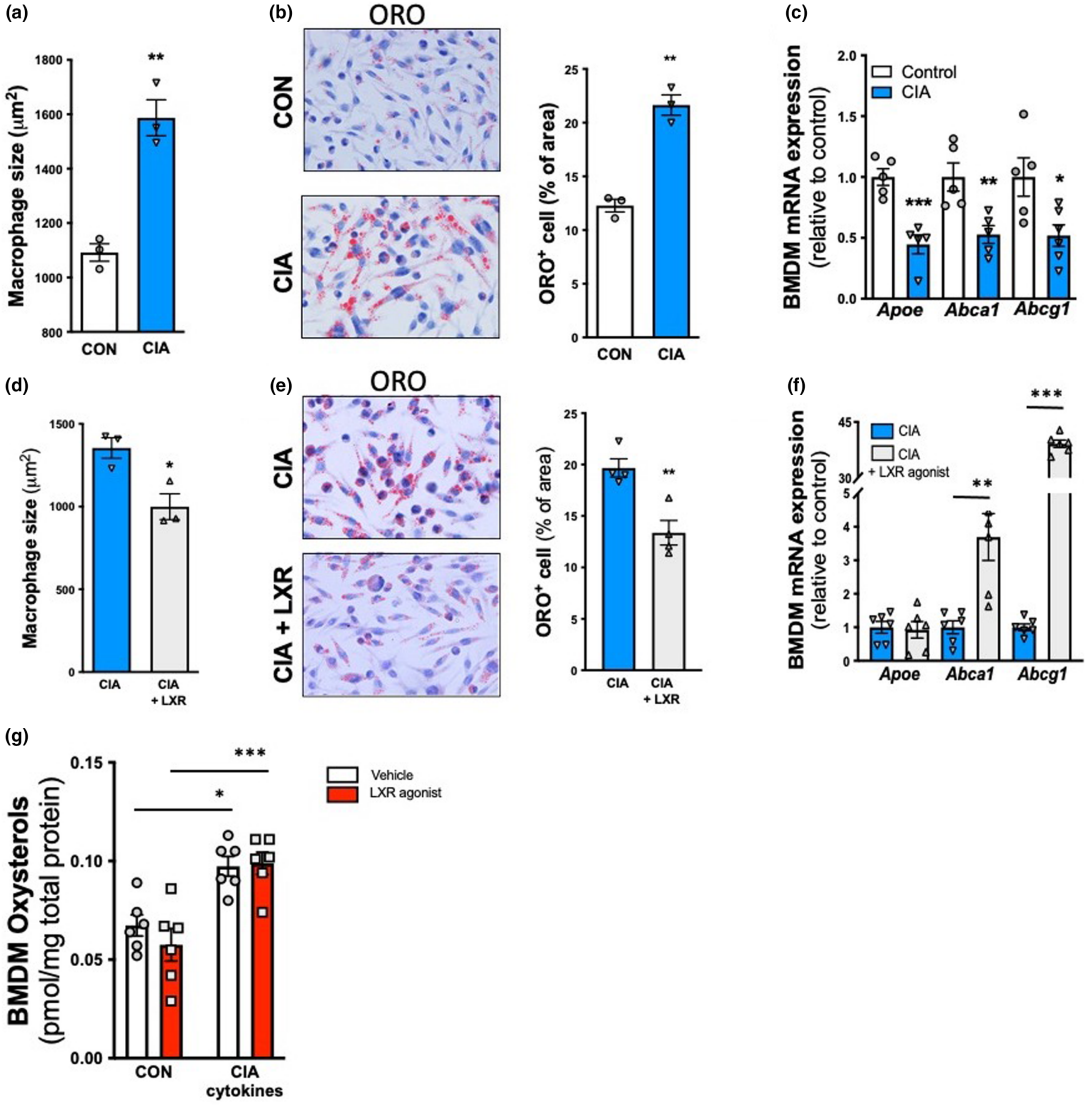
Arthritic serum promotes lipid‐laden macrophages, which is reversed with an LXR agonist. Bone marrow‐derived macrophages (BMDMs) were treated with pooled serum from either control or arthritic (RA) serum, in the presence and absence of LXR agonist T0901317. **(a, d)** BMDM size, **(b, e)** Oil Red O lipid content and **(c, f)** gene expression. **(g)** Total oxysterol levels in BMDMs treated with RA‐associated cytokines (TNF 2.5 ng mL^−1^, GM‐CSF 2 ng mL^−1^, IL‐1β 7.5 ng mL^−1^ and IL‐6100 ng mL^−1^), in the presence and absence of T0901317 (3 μM) were quantified by mass spectrometry lipidomics. *n* = 3–6 biological replicates using serum from *n* = 3–6 mice, 1 experiment. *P < 0.05, **P < 0.01, ***P < 0.001 as indicated. All data are mean ± SEM.

Therefore, we investigated whether inflammatory arthritis directly promotes foam cell formation. To do this, we incubated bone marrow‐derived macrophages (BMDMs) with pooled serum from either control or arthritic (collagen‐induced arthritis; CIA) mice and assessed both cellular lipid accumulation and alterations in mRNA expression of the cholesterol efflux transporters. BMDMs treated with CIA serum were larger and accumulated more lipid compared with control, and the cholesterol efflux genes *Apoe*, *Abcg1* and *Abcg1* were dramatically reduced, indicating reduced cholesterol efflux and increased foam cell development (Figure 3a–c). Further analysis of cholesterol metabolism genes revealed an increase in *Ldlr* and *HmgCoR* expression and decreased *Lxra, Cyp27a* and *Srebf1* expression (Supplementary figure 1a and b). Collectively, these data reveal that increased lipid accumulation in BMDMs treated with serum of arthritic mice is likely driven by a combination of reduced cholesterol efflux, increased lipid anabolism and decreased lipid catabolism.

Having confirmed a foam cell‐like phenotype in macrophages exposed to arthritic serum, we next explored whether these macrophages would be responsive to the LXR agonist. Indeed, arthritic serum and T0901317 administration reduced macrophage size, Oil Red O (ORO) lipid content and unlike the HSPCs, dramatically increased *Abca1* and *Abcg1*, but not *Apoe*, gene expression, compared with BMDMs treated with arthritic serum alone (Figure 3d–f). This suggests that pharmacologically activating LXR is able to restore the cholesterol metabolism defects on BMDMs exposed to serum of arthritic mice, thus reducing lipid accumulation in macrophages.

### Endogenous LXR dysfunction in BMDMs is likely driven by a failure of oxysterol signalling

Physiologically, LXR is activated by oxysterols which are generated when the cell senses increased cholesterol. Therefore, to assess whether the defect in LXR signalling is because of a failure of oxysterol production or signalling, we performed lipidomics on BMDMs treated with a cocktail of inflammatory cytokines associated with rheumatoid arthritis (TNF, GM‐CSF, IL‐1β and IL‐6). Indeed, macrophages treated with inflammatory cytokines increased in cellular oxysterol levels (Figure 3g), which is expected given the increased lipid loading in these macrophages. LXR activation with T0901317 did not alter cellular oxysterol levels (Figure 3g).

Liver X receptor dimerises with retinoid X receptor (RXR) to form the LXR/RXR complex upon oxysterol‐induced activation.^37^, ^38^ This complex then binds directly to LXR response elements on target genes. The regulation of LXR/RXR activation is controlled by co‐repressors and co‐activators of this heterodimeric complex.^38^, ^39^ In basal conditions, LXR/RXR is bound to co‐repressors such as silencing mediator of retinoic acid and thyroid hormone receptor (SMRT) or nuclear co‐repressor 1 (NCOR1).^38^ Following a conformational shape change during LXR activation, co‐activators such as nuclear co‐activator‐1 (NCOA1) or activating signal co‐integrator 2 (ASC2) are recruited to initiate transcription of target genes.^38^ Gene expression analysis of these accessory proteins did not reveal any differences in BMDMs exposed to arthritic serum (data not shown).

Collectively, these data show that BMDMs treated with inflammatory cytokines are able to sense increased cellular cholesterol accumulation and increase oxysterol production. However, there is a failure by the oxysterols to stimulate LXR activity. Administration of T0901317 may bypass this pathway, to pharmacologically activate the LXR and reduce foam cell formation in BMDMs.

### LXR activation increases ABCA1 in atherosclerotic lesions of arthritic mice

**Figure 4:**
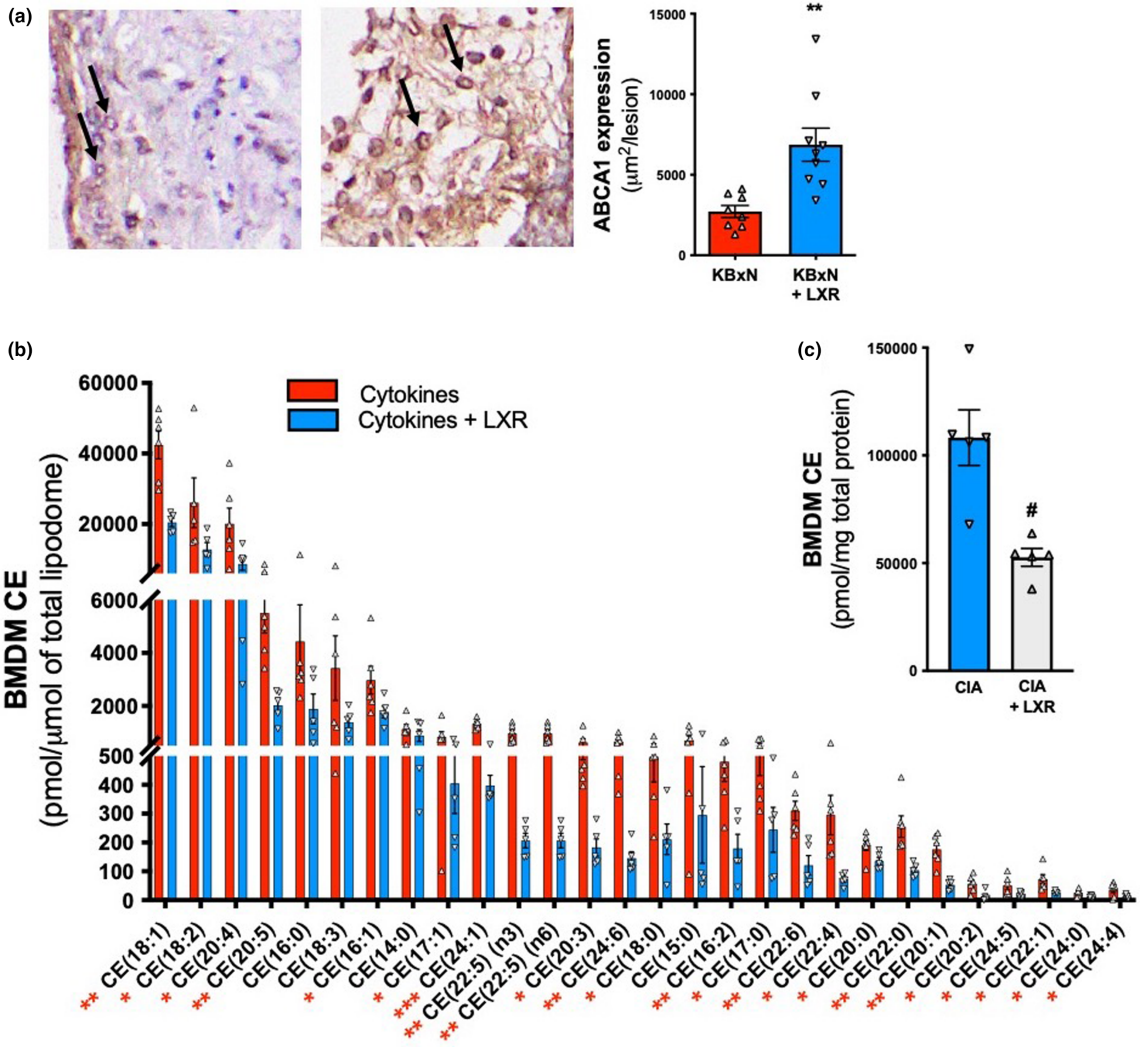
LXR agonist increases ABCA1 abundance in lesions of arthritic mice, and reduced cholesteryl esters in BMDMs treated with T0901317. **(a)** ABCA1 protein expression in the atherosclerotic plaques from the cohort of mice in Figure 1 was determined via immunohistochemistry. *n* = 8 and 9 mice/group, in 1 cohort. **(b, c)** Mass spectrometry lipidomics was employed to measure cholesterol ester (CE) levels in BMDMs treated with **(b)** RA‐associated cytokines (TNF 2.5 ng mL^−1^, GM‐CSF 2 ng mL^−1^, IL‐1β 7.5 ng mL^−1^ and IL‐6100 ng mL^−1^) or **(c)** CIA serum in the presence and absence of the T0901317 (3 μM). *n* = 6 (cytokine) and 5 (cytokine +LXR) biological replicates, 1 experiment. *P < 0.05, **P < 0.01 as indicated. All data are mean ± SEM.

We next sought to confirm T0901317‐induced LXR activation in lesional macrophages in the atherosclerotic regression study (Figure 1). This was achieved by assessing ABCA1 protein levels using immunohistochemistry. In line with the *in vitro* data, we observed an almost threefold increase of ABCA1 protein in lesions from arthritic mice treated with T0901317, compared with vehicle‐treated mice with RA (Figure 4a). This suggests that ABCA1‐mediated cholesterol was restored in plaque macrophages, contributing to LXR‐induced lesion regression in arthritis.

### T0901317 promotes clearance of cellular cholesteryl esters from BMDM foam cells treated with inflammatory cytokines

The hallmark feature of macrophage foam cell formation is the creation of cellular lipid droplets that store the excess lipid. LDL cholesterol is taken up by the cell and degraded into cholesteryl esters (CEs), which are then further modified in lysosomes in order to be stored in lipid droplets.^40^ Additionally, cytoplasmic clearance of CEs occurs via effective ABCA1‐dependent cholesterol efflux. Therefore, in order to confirm that T0901317 can reduce foam cell formation in macrophages exposed to inflammatory cytokines, we treated BMDMs with inflammatory cytokines (TNF, GM‐CSF, IL‐1β and IL‐6) in the presence and absence of T0901317 and performed lipidomics to assess CE levels. We observed a reduction in 24 of the 28 CE lipid species detected (Figure 4b). We also cultured BMDMs in media containing serum from CIA mice with or without T0901317 and observed a dramatic reduction of CE levels in BMDMs treated with the LXR agonist (Figure 4c). Collectively, these data, along with the increase in plaque macrophage ABCA1 expression, suggest that LXR activation can reduce foam cell formation by promoting the efflux of CEs via an ABCA1‐dependent mechanism.

### LXR activation reduces joint, systemic and plaque inflammation

**Figure 5:**
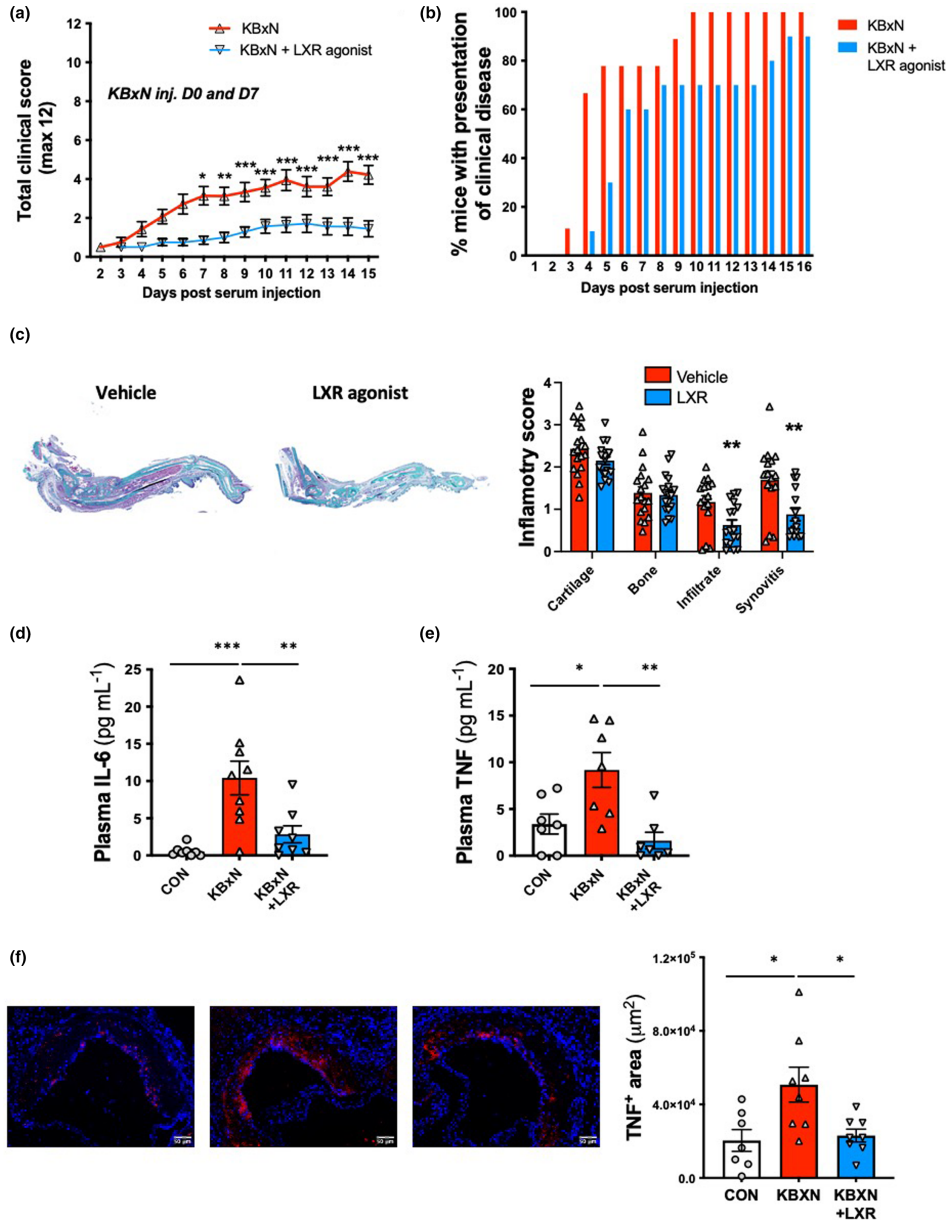
LXR agonist T0901317 reduces arthritic clinical score along with joint, systemic and plaque inflammation. Arthritic parameters were quantified in the *Ldlr* ^*−/−*^ from Figure 1 **(a)** Arthritic clinical scores and **(b)** percentage of mice that presented with clinical arthritis. **(c)** Histological assessment of arthritic 2 feet per animal, as determined by Safranin‐O staining. **(d, e)** Plasma cytokine levels. **(f)** Representative images and quantification of atherosclerotic plaque TNF (scale bar = 50 μm). *n* = 7–9 mice, 1 cohort. *P < 0.05, **P < 0.01, ***P < 0.001 as indicated. All data are mean ± SEM.

To assess the role of LXR activation in the pathogenesis of inflammatory arthritis, we compared the severity of the arthritis of vehicle‐ and T0901317‐treated mice both clinically and histologically. Arthritic mice developed 2 weeks of persistent inflammatory arthritis (Figure 5a). However, mice treated with T0901317 had markedly reduced clinical scores and also exhibited a delayed onset of joint disease (Figure 5b). Subsequently, ankle joints were processed for histology, and sections were graded by a blinded investigator. Arthritic mice displayed severe cartilage damage, moderate bone destruction, infiltration of immune cells and synovial hyperplasia (Figure 5c). Importantly, LXR activation reduced both immune cell infiltrate and synovitis, suggesting that T0901317 reduced leukocyte‐driven joint inflammation (Figure 5c). We also confirmed the antiarthritic effects of LXR activation in the widely used CIA model. Administration of T0901317 also reduced arthritis in the CIA mice, which was also independent of changes in circulating monocytes and HSPC cholesterol efflux gene expression (Supplementary figure 2). As expected, systemic inflammation (as assessed by plasma IL‐6 and TNF levels) was elevated in the arthritic mice (Figure 5d and e). Consistent with reduced joint inflammation, these cytokines were significantly reduced when the mice received the LXR agonist (Figure 5d and e). We also observed increased TNF in the plaques of the arthritic mice, which was significantly reduced by LXR treatment (Figure 5f). Taken together, LXR appeared to reduce both systemic and joint inflammation as well as improving atherosclerotic lesion regression in arthritic mice.

## Discussion

**Figure 6:**
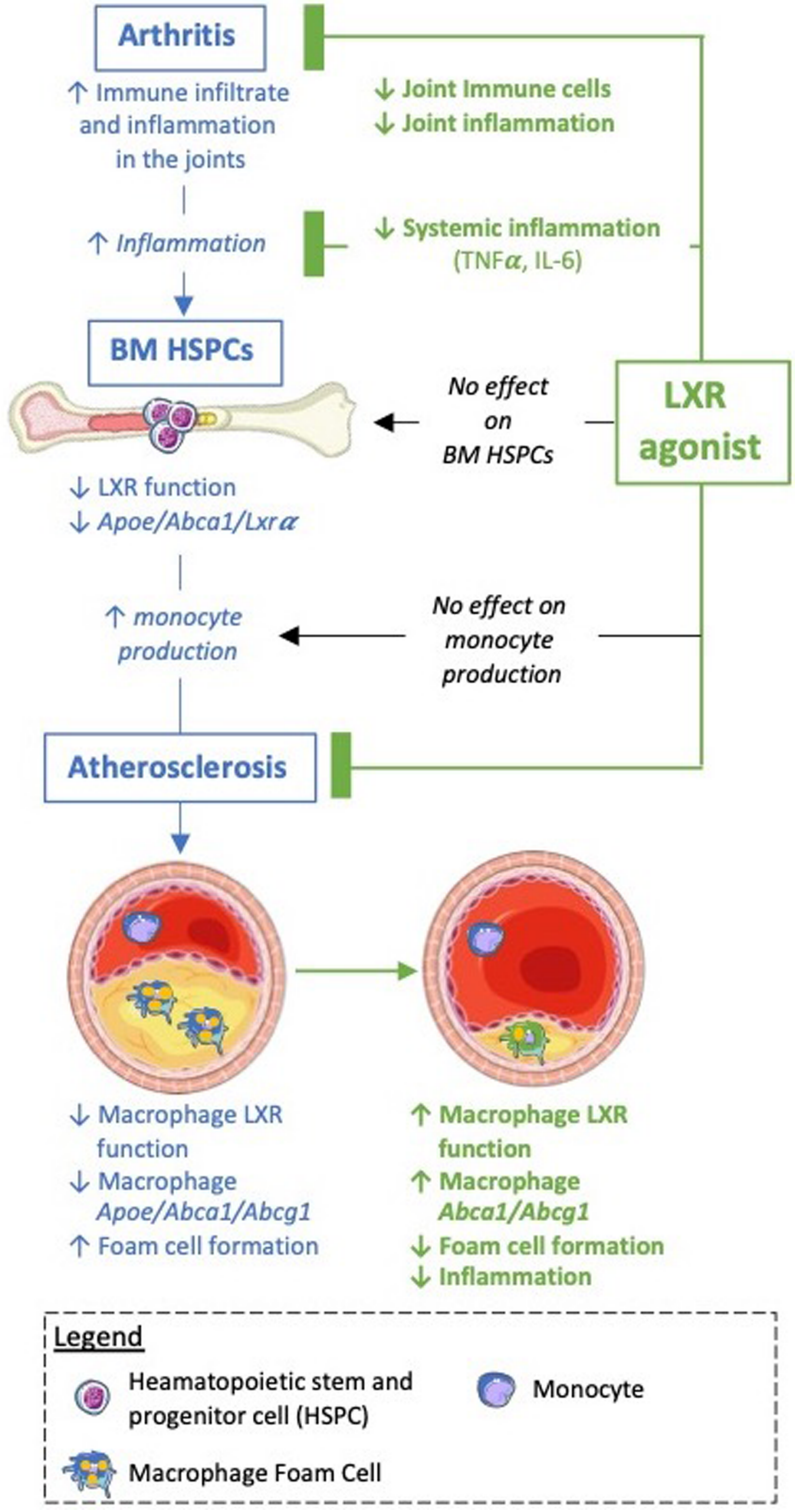
Overview of anti‐inflammatory effect of LXR activation in arthritic mice and how this reduces atherosclerosis.

Rheumatoid arthritis is an inflammatory autoimmune disorder that increases the risk premature CV mortality. We have previously shown that arthritis induces cellular cholesterol defects, which are associated with impaired atherosclerotic lesion regression.^14^ In this study, we revealed that LXR activation induces atherosclerotic lesion regression in arthritic mice, independently of circulating plasma cholesterol levels. LXR agonism reduced inflammatory cell infiltrate and lipid accumulation in the atherosclerotic lesions, which was accompanied with an increase in lesion ABCA1 expression and reduced foam cell formation (Figure 6). Moreover, LXR activation reduced the onset and severity in two experimental models of joint inflammation, as well as reduced systemic inflammation (Figure 6). Thus, this study suggests that restoring efficient cholesterol handling is a potential approach to reduce arthritis disease severity, systemic inflammation and the exacerbated CVD that occurs in human RA.

We have previously shown that RA impairs atherosclerotic lesion regression and promotes an unstable plaque phenotype, which was associated with defects in cellular cholesterol metabolism.^14^ In this study, we found that administering the LXR agonist T0901317 promoted atherosclerotic plaque regression in the setting of murine arthritis. This effect was associated with positive remodelling of the atherosclerotic plaques. Most notably, we observed a reduction in lesional macrophage foam cell abundance. We hypothesised that administration of the LXR agonist would improve cellular cholesterol metabolism in HSPCs, by inducing the expression of the HSPC intrinsic cholesterol efflux system, as we have shown previously.^15^ This would remove excess cellular cholesterol in the HSPCs and limit their proliferation, and hence the production of monocytes, which are important in driving atherosclerosis. To our surprise, even though the arthritic mice treated with the LXR agonist had a milder arthritic burden and smaller atherosclerotic lesions, the HSPCs did not respond to the LXR activator (Figure 6). This finding suggests that some cells in the arthritic mice remained LXR responsive (e.g. macrophages), while others (i.e. HSPCs) were not. Why this occurred is unknown but could be as a result of the marked hypercholesterolemia associated with LXR activation in the arthritic mice. Alternatively, there could be residual inflammation that was not suppressed in the LXR‐treated mice. For example, while circulating IL‐1𝛽 was below the detection limits (data not shown), we recently found that IL‐1𝛽 was important in driving myelopoiesis in K/BxN‐induced arthritic mice.^41^

To explain the positive effects of the LXR agonist on lesion regression, we examined the plaque macrophages. Modelling this *in vitro*, we found that serum from arthritic mice stimulated atherosclerotic lesion formation, evidenced by augmented macrophage lipid accumulation and downregulation of the cholesterol efflux genes *Abca1*, *Abcg1* and *Apoe*, and increased cholesterol synthesis genes *Ldlr* and *HmgCoR*. In keeping with this, Voloshyna and colleagues have previously documented enhanced foam cell formation using plasma from patients with RA.^21^ Furthermore, oxysterols (oxidised derivatives of cholesterol) that activate LXR to protect the cells from excessive cholesterol accumulation^39^ were upregulated in lipid‐laden BMDMs exposed to inflammatory cytokines (TNF and IL‐6) associated with RA. Taken together, these data suggest a clear defect in LXR signalling in the setting of inflammation.

Interestingly, we found this phenotype was rescued with LXR activation, suggesting that macrophage LXR could still be activated with synthetic stimulators to reduce foam cell formation (Figure 6). This was confirmed by enhanced ABCA1 expression in the lesions of the mice treated with the LXR agonist and specifically reduced CE accumulation in BMDMs expose to inflammatory cytokines in the presence of LXR activation. There is evidence that regulating cellular cholesterol improves CVD in RA. Rosuvastatin has been shown to promote carotid plaque atherosclerotic regression in patients with RA, independent of its LDL‐lowering effects.^42^ This suggests that statin‐induced inhibition of cellular cholesterol biosynthesis could be important in reducing atherosclerotic burden. Additionally, it has been documented that cellular lipid, or its metabolites, can activate toll‐like receptors (TLRs) to impact macrophage biology, including inflammatory responses.^43^, ^44^ Taken together, this supports a role for regulating cellular cholesterol levels in the management of CVD in RA. Whether blocking intrinsic cholesterol biosynthesis would also inhibit myelopoiesis would be interesting, especially since administration of rHDL can suppress monocyte production in experimental RA and in genetic models of defective cholesterol efflux.

Targeting LXR may have anti‐inflammatory and antiatherogenic effects. Indeed, we observed a reduction in plaque TNF in the arthritic mice rerated with the LXR agonist. LXR activation can dampen inflammation by inducing transrepression of NFκB genes (including TNF),^45^ which can also contribute to lesion regression. Macrophages lacking *Lxr*, or the transporters *Abca1/Abcg1*, exhibit persistent TLR activation and an exacerbated inflammatory response evidenced by increased LPS‐induced *inos*, *Il‐1β* and *mcp‐1* gene expression, which can be reduced by LXR agonism.^18^, ^39^, ^46^ The most well‐characterised mechanism for this is the indirect repression of inflammatory genes, which is thought to occur by SUMOylation of LXR by SUMO2/3, promoting an interaction with the repressor N‐CoR with NFκB, and in turn, preventing transcription of NFκB target genes.^39^, ^47^, ^48^ LXR‐induced transrepression can diminish inflammatory mediators such as MCP‐1, IL‐6, ICAM1, E‐selectin and MMP9, which are essential in plaque progression and vulnerability.^25^, ^49^, ^50^ Moreover, reduction in these inflammatory cytokines and chemokines not only improves the plaque microenvironment but can also reduce monocyte recruitment and entry into atherosclerotic lesions. Supporting this hypothesis, LXR agonism inhibited NFκB activity in endothelial cells, suppressing inflammation and reducing monocyte adhesion.^51^ LXR activation clearly suppresses an array of inflammatory processes, which results in less monocyte‐derived plaque macrophages, reduced macrophage‐driven inflammation and ultimately, lesion regression.

Taken together, we show that the LXR agonist T0901317 rescues impaired atherosclerotic lesion regression in inflammatory arthritis. This was independent of cholesterol levels or circulating leukocytes, but rather because of enhanced cholesterol efflux transporter expression and reduced foam cell development in atherosclerotic lesions. LXR agonism could reduce inflammatory pathways in multiple cell types involved in atherosclerosis. Given the undesirable lipogenic effects of LXR stimulation, these findings contribute to a large body of evidence that site‐targeted delivery of LXR agonists could be beneficial in reducing plaque burden. Moreover, this study supports the notion that restoring efficient cholesterol handling in diseases with systemic inflammation represents a novel approach to reduce both disease severity and exacerbated CVD in these patients.

## Methods

### Animals

All experiments were approved by the Alfred Medical Research Education Precinct (AMREP) animal ethics committee. WT (C57BL/6J) and *Ldlr* ^*−/−*^ mice were purchased from Jackson Laboratories and colonies were maintained at AMREP animal facilities. DBA mice were purchased from the Walter and Eliza Hall Institute of Medical Research (WEHI) facility (Kew, Australia). All mice were housed in a normal light and dark cycle and had access to food and water *ad libitum*. Mice were fed a normal chow diet unless stated otherwise.

### Atherosclerotic regression study

#### Experimental design

To perform the atherosclerotic regression experiments, female *Ldlr* ^*−/−*^ mice were fed a western‐type diet (WTD; 22% Fat, 0.15% cholesterol; catalogue #SF00‐219, Specialty Feeds) for 14 weeks. To induce lesion regression, *Ldlr* ^*−/−*^ were then switched to a normal chow diet for 3 weeks. Mice undergoing regression were divided into three groups; (1) control, (2) K/BxN serum transfer arthritis (2× intraperitoneal injection of K/BxN serum; 100 μL per injection) and received vehicle (30% DMSO‐saline, I.P.) or (3) rendered arthritic using K/BxN serum and were administered LXR agonist T0901317 daily for 2 weeks (25 mg kg^−1^, I.P.).

#### Lesion characteristics

Atherosclerotic lesion size was determined by haematoxylin and eosin staining, lipid content was determined by Oil Red O (ORO) staining, macrophage abundance was determined by CD68 immunohistochemistry staining, and collagen content was determined by Picrosirius staining (see below for details).

#### Stem cell‐enriched BM

Bone marrow was harvested from the tibia and femur, using Iscove's Modified Dulbecco's Medium (IMDM). BM was incubated with 10% foetal calf serum (FCS) and stem cell factor (SCF, 100 ng mL^−1^) in IMDM for 2 h to enrich for HSPC populations. The supernatant was acquired, washed with PBS and processed for RNA extraction using TRIzol, and cDNA synthesised using Bioline Tetro cDNA synthesis Kit (catalogue number BIO‐65043), both as per manufacturer's instructions.

### Short‐term LXR agonist experiment in arthritic C57bl/6 mice

#### Experimental design

Male C57bl/6J mice (8–10 weeks of age) were either left as nonarthritic controls or received 1 intraperitoneal injection of K/BxN serum (100 μL) to induce the K/BxN serum transfer model of arthritis. A subset of mice in each group subsequently were administered the LXR agonist T0901317 (25 mg kg^−1^, I.P.), while the remaining mice received the vehicle control (30% DMSO‐saline, I.P.) for three consecutive days before sacrifice.

#### Bone marrow haematopoietic stem and progenitor cells (HSPCs) isolation

BM HSPCs were identified as stated in flow cytometry (see below) and isolated using fluorescence‐activated cell sorting (FACS) into RLT lysis buffer from the Qiagen kit. After collection, RNA was extracted using the Qiagen RNA Isolation Kit (RNeasy Mini Kit; catalogue number 74104) and cDNA synthesised using the Bioline Tetro cDNA synthesis Kit (catalogue number BIO‐65043), as per the manufacturer's instructions.

### Atherosclerotic lesions and characteristics

Aortic atherosclerotic lesions in the aortic root were analysed on 6‐μm frozen sections.

#### Lesion size

Sections were fixed (4 min, 10% neutral‐buffered formalin), washed in PBS (4 min), stained in Mayer's Haematoxylin (15 min) and washed with running tap water before blueing in Scott's tap water for 30 s. The slides were then put in 95% ethanol (10 dips), stained in buffered alcoholic eosin (8 min), dehydrated in absolute ethanol and cleared with xylene, and coverslips were mounted using depex. Sections were imaged on the Olympus FSX100 microscope 4.2× magnification, and images were analysed using Adobe Photoshop CC.

#### Lipid content

Sectioned lesions were fixed in 10% buffered formalin (4 min), washed in PBS (4 min) and dipped in 60% isopropanol before staining in 60% ORO working solution (2 h, stock solution: 1% ORO powder in isopropanol). The slides were then washed in 60% isopropanol and distilled water. Sections were stained in Mayer's Haematoxylin (4 min), washed in tap and distilled water (3 min each) and mounted with aquamount. Sections were imaged on the Olympus FSX100 microscope 4.2× magnification, and images were analysed using Adobe Photoshop CC.

#### Macrophage abundance

Thawed sections were fixed with paraformaldehyde (4%, 20 min), washed in PBS (2 × 5 min), incubated in prechilled 3% H_2_O_2_ in methanol (20 min) and then washed in PBS (2 × 5 min). Each section was blocked with normal goat serum (NGS, 10%, 30 min), incubated with AVIDIN blocking solution (15 min), rinsed in PBS and then incubated with rat anti‐mouse CD68 primary antibody (1:200, 5% NGS, 4°C) overnight. The slides were then washed in PBS (2 × 5 min) before being incubated with the secondary antibody (1:100, 5% NGS, 30 min). Next, the sections were washed in PBS (2 × 5 min) and incubated with ABC avidin/biotin complex (30 min) and DAB solution. Staining reaction was terminated with distilled water. The sections were counterstained with Mayer Haematoxylin for 15 s and rinsed in tap water before blueing in Scott's tap water and washing in tap water. Finally, slides were dehydrated in ethanol (95% 3 min, 100% 3 × 3 min), cleared in xylene (2 × 5 min) and mounted with depex. Sections were imaged on the Olympus FSX100 microscope 4.2× magnification, and images were analysed using Adobe Photoshop CC.

#### Collagen

Sections were thawed and fixed in prechilled acetone (15 min), washed in PBS (2 × 5 min), stained in 0.1% Sirius red F3BA (1 h) and then washed in 0.01 m HCl (2 min). Subsequently, the slides were then dehydrated in alcohol (95%, 5 min; 100%, 2 × 5 min), cleared in xylene (2 × 5 min) and mounted with depex. Sections were imaged on Olympus BX61 microscope under bright‐field and polarised light 4.2× magnification, and images were analysed using Adobe Photoshop CC.

#### ABCA1

ABCA1 immunohistochemistry was performed with the same protocol as with CD68 staining, with the exception of the primary and secondary antibodies. Rabbit anti‐mouse ABCA1 primary antibody (#400‐105, Novus) and goat anti‐rabbit secondary were utilised for these experiments.

#### TNF

Frozen sections were fixed with 4% PFA for 10 min and blocked overnight at 4°C with 1% BSA/0.2% Triton X‐100/10% Donkey Serum/PBS. TNF alpha Monoclonal Antibody (1:50, Thermo Fisher Scientific) was incubated overnight at 4°C. Following primary antibody staining, sections were washed and incubated with Donkey anti‐Rat IgG (H + L) AF647 (1:200, ThermoFisher Scientific) and DAPI (1 μg mL^−1^) for 30 min at room temperature. Sections were then washed and mounted using Prolong Diamond Mounting Solution (Thermo Fisher Scientific). Sections were imaged using an Olympus BX‐71 microscope and analysed using Image J.

### Induction of collagen induced arthritis

Collagen‐induced arthritis was induced in 8–10‐week‐old male DBA mice as previously described.^14^ Briefly, mice were immunised with an intradermal injection of chicken type II collagen (CII) (2 mg mL^−1^; Sigma‐Aldrich) emulsified in a 1:1 volume of complete Freund's adjuvant (CFA) (containing 5 mg mL^−1^ heat‐killed *Mycobacterium tuberculosis H37RA*, Difco Laboratories, Detroit, MI, USA) on Day 0 and Day 21.

### Clinical joint scoring of arthritic mice

Clinical arthritis severity was graded by scoring each limb on a scale from 0 to 3, where 0 = no erythema and swelling; 1 = mild erythema and swelling confined to the ankle, wrist or digits; 2 = mild erythema and swelling extending from the ankle to the mid‐foot, 3 = moderate erythema and swelling.

### Histology of arthritic paws

The ankle joint was evaluated histologically. Paws of mice were fixed in 10% neutral‐buffered formalin, embedded in paraffin, sectioned at 7 μm and stained with Safranin‐O, according to standard practice. Histological analysis was performed blinded on serial joint sections for synovitis, cell influx, cartilage damage and bone degradation. Histology scores for each parameter are as follows: 0 = normal, 1 = mild, 2 = moderate, 3 = severe.

### Mouse total cholesterol

Total serum cholesterol levels were measured from plasma of mice using the Cholesterol E kit (Wako Diagnostics) per the manufacturer's instructions.

### Flow cytometry

#### Blood monocytes

Monocytes and monocyte subsets were identified using flow cytometry as previously described.^14^ Blood was collected via tail bleeding and collected into EDTA tubes, which were immediately incubated on ice. All subsequent steps were performed on ice. Red blood cells were lysed (BD pharm Lyse; BD Biosciences), and WBCs were centrifuged, washed and resuspended in HBSS (0.1% BSA w/v, 5 mM EDTA). Cells were stained with a cocktail of antibodies against CD45‐PB, Ly6‐C/G‐PerCP‐Cy5.5 (BD Biosciences) and CD115‐APC (eBioscience). Monocytes were identified as CD45^hi^CD115^hi^ and further subdivided into Ly6‐C^hi^ and Ly6‐C^lo^ (Supplementary figure 3). Samples were run on the Canto II or LSR Fortessa and analysed using FlowJo.

#### Bone marrow haematopoietic stem and progenitor cells (HSPCs)

Haematopoietic stem and progenitor cells were identified using flow cytometry as previously described.^14^ BM was harvested from the femurs and tibias. BM was lysed with RBC lysis buffer (BD pharm Lyse; BD Biosciences), and the remaining cells were centrifuged, washed and resuspended in HBSS (0.1% BSA w/v, 5 mM EDTA) buffer. Briefly, a cocktail of antibodies to lineage (lin) committed cells (CD45R, CD19, CD11b, CD3e, TER‐119, CD2, CD8, CD4, and Ly‐6G; all FITC; eBioscience) and stem cell markers Sca1‐Pacific Blue and ckit‐APC‐Cy7. HSPCs were identified as lin^−^Sca1^+^ckit^+^.

### *In vitro* BMDM experiments

#### Serum isolation

Serum was collected by collecting the blood with no anticoagulants, and the blood was allowed to clot for 30 min at RT. Blood was then centrifuged at 2000 *g* for 10 min (4°C), and the supernatant collected.

#### BMDM differentiation and treatment

Bone marrow from male WT DBA mice was harvested from the tibia and femur, using RPMI‐1640 Medium. Cells were resuspended in L‐cell media (15% FBS, 1× Pen/Step antibodies, 20% L‐cell media in RPMI‐glutamax) at a concentration of 10^6^ BM cells per mL media, for 7 days to induce differentiation of BM‐derived macrophages (BMDMs). BMDMs were treated with 15% serum in RPMI‐glutamax media from either control or arthritic mice (from collagen‐induced arthritis; CIA). Separately, BMDMs were treated with CIA serum and T0901317 (3 μM), or CIA serum and vehicle (DMSO). Lipid abundance and gene expression were assessed following 18‐h and 6‐h incubation, respectively.

#### Gene expression

Samples were then washed once in PBS and processed for RNA extraction using TRIzol reagent, and cDNA using Tetro cDNA kit (Bioline), as per manufacturer's instructions.

#### Lipid abundance

Media was removed, and cells were fixed in 4% PFA (room temperature, 20 min) and them washed once with PBS. Cells were exposed to isopropanol (60%, 30 s), after which they were stained with Oil Red O working solution, and washed once more with isopropanol (60%, 15 s). Subsequently, cells were washed 3 times with PBS, counterstained with Mayers (20 s) and then washed with tap water. Cells were fixed with Aquamount and allowed to dry. Cells were imaged using the BX43 microscope (40× objective) and analysed using Adobe Photoshop CC.

### Lipidomics—general lipid extraction and analysis

BMDMs were scraped, collected in 200 μL of ice‐cold PBS and sonicated (S‐4000; Misonix) and protein concentrations determined (Thermo Fisher Scientific, #23225) to allow for data normalisation during analysis. Samples were then dried overnight in a Speedvac (Thermo Scientific) in preparation for lipid extraction. Lipids were extracted using a single‐phase chloroform/methanol extraction as described previously with modification for cultured cells.^52^

Liquid chromatography–tandem mass spectrometry was performed according to previously published methods, with slight modification for cultured cells.^53^ Cellular extracts were analysed using a 4000 Qtrap mass spectrometer (AB Sciex) with an Agilent 1290 series HPLC and a ZORBAX eclipse plus C18 column (2.1 × 100 mm 1.8 μm, Agilent) with the thermostat set at 60°C. Mass spectrometry analysis was performed using dynamic scheduled multiple reaction monitoring in positive ion mode; transitions, internal standards and conditions have been previously reported.^52^ Cholesteryl esters were monitored as their ammoniated adduct, with a characteristic 369 product ion. Data were analysed in MultiQuant 2.1.1 (AB Sciex) software. Lipid abundances were determined by normalising the area under the chromatogram for each lipid species against the corresponding internal standard.

### Lipidomics—oxysterol extraction and analysis

After lipid extraction as described above, dried‐down lipid extracts were derivatised using a method adapted from Griffiths *et al*.^54^ To each sample, 200 μL of PBS with 96 unit mL^−1^ of cholesterol oxidase was added. Samples were incubated at 37°C for 1 h and 30 min with frequent vortexing. Afterwards, 500 μL of methanol with 10 mM Girard's Reagent P was added with 20 μL of acetic acid. Samples were then left overnight at room temperature on a rotary shaker. Derivatisation was stopped through the addition of 500 μL acetone. Samples were vortexed and left to sit at room temperature for a further 15 min. Samples were then completely dried on the SpeedVAC and reconstituted in 200 μL of butanol:methanol 1:1 with 10 mM ammonium formate and sonicated for a further 10 min. Samples were then centrifuged, and supernatants were transferred to sample vials with glass inserts for mass spectrometry analysis.

Samples were analysed on an Agilent 6490 QQQ mass spectrometer in conjunction with an Agilent 1290 HPLC system using a ZORBAX eclipse plus C18 column (2.1 × 100 mm 1.8 μm, Agilent). The mass spectrometry conditions used were as follows: positive ionisation mode, gas temperature, 150°C, gas flow rate 17 L min^−1^, nebuliser 20 psi, Sheath gas temperature 200°C, capillary voltage 3500 V and sheath gas flow 10 L min^−1^. Isolation widths for Q1 and Q3 were set to ‘unit’ resolution (0.7 amu). A specific transition Q1–534.4 *m/z*, Q3–455.3 *m/z* was used to confirm oxysterol species that were derivatised by the GP reagent.

### ELISAs

TNF and IL‐6 ELISAs were performed as per the manufacturer's instructions (ThermoFisher).

### Gene expression (real‐time PCR)

All mRNA expression was detected using fast SYBR Green primers, and the gene of interest was controlled using the housekeeper *Gapdh*. Primer sequences are in the online supplement (Supplementary table 1).

### Statistics

Statistical significance was determined by the two‐tailed parametric Student's *t*‐test (to compare two groups) or by one‐way ANOVA with the Bonferroni multiple comparisons test (to compare three groups) using GraphPad Prism. A *P*‐value < 0.05 was considered significant. Data are expressed as mean ± SEM.

## Author contributions

**Dragana Dragoljevic:** Conceptualization; data curation; formal analysis; investigation; methodology; project administration; supervision; validation; visualization; writing – original draft; writing – review and editing. **Man Kit Sam Lee:** Conceptualization; data curation; formal analysis; investigation; methodology; writing – original draft; writing – review and editing. **Gerard Pernes:** Data curation; formal analysis; methodology; writing – review and editing. **Pooranee K Morgan:** Data curation; formal analysis; investigation; methodology; writing – review and editing. **Cynthia Louis:** Data curation; formal analysis; investigation; methodology; visualization; writing – review and editing. **Waled Shihata:** Data curation; formal analysis; investigation; methodology; writing – review and editing. **Kevin Huynh:** Data curation; formal analysis; investigation; methodology; writing – review and editing. **Arina A Kochetkova:** Investigation; writing – review and editing. **Patrick W Bell:** Investigation; writing – review and editing. **Natalie A Mellett:** Data curation; formal analysis; investigation; methodology; writing – review and editing. **Peter J Meikle:** Investigation; methodology; supervision; writing – review and editing. **Graeme I Lancaster:** Investigation; methodology; project administration; supervision; writing – review and editing. **Michael J Kraakman:** Data curation; formal analysis; investigation; methodology; project administration; supervision; writing – review and editing. **Prabhakara R Nagareddy:** Conceptualization; investigation; project administration; supervision; writing – review and editing. **Beatriz Y Hanaoka:** Conceptualization; project administration; supervision; writing – original draft; writing – review and editing. **Ian Wicks:** Conceptualization; investigation; methodology; project administration; resources; supervision; writing – original draft; writing – review and editing. **Andrew Murphy:** Conceptualization; data curation; formal analysis; funding acquisition; investigation; methodology; project administration; resources; supervision; writing – original draft; writing – review and editing.

## Conflict of interest

The authors declare no conflicts of interest.

## Supporting information


Supplementary figure 1
Supplementary figure 2
Supplementary figure 3
Supplementary table 1

## Data Availability

The datasets generated during and/or analysed during the current study are available from the corresponding author on reasonable request.
