# Supplementary material for: Administration of an LXR agonist promotes atherosclerotic lesion remodelling in murine inflammatory arthritis
Source: Clin Transl Immunology. 2023 Apr 18;12(4):e1446. doi: 10.1002/cti2.1446 (PMC10113696; doi:10.1002/cti2.1446)
Supplement: Supplementary file 1 — Supplementary figure 1 Supplementary figure 2 Supplementary figure 3 Supplementary table 1 [file CTI2-12-e1446-s001.pdf]

SUPPORTING INFORMATION

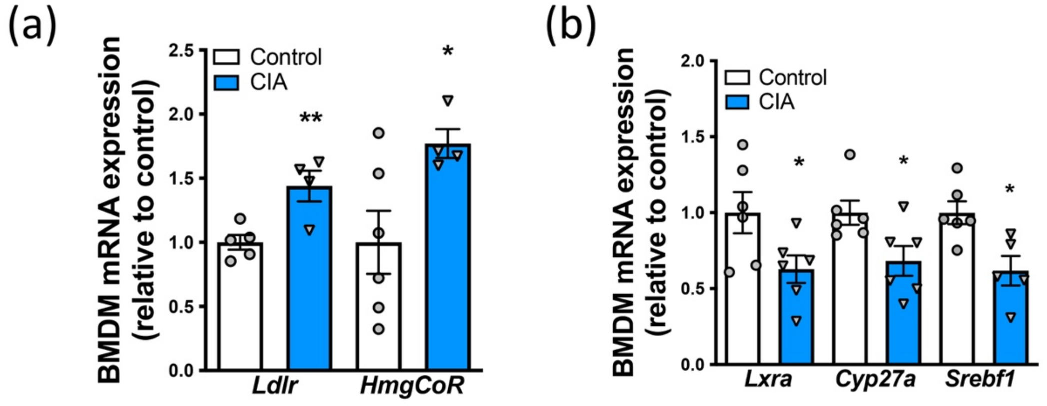

Supplementary Figure 1. Bone marrow derived macrophages (BMDMs) were treated with pooled serum from either control or arthritic (IA) serum, and gene expression was assessed. n = 3-6 biological replicates, 1 experiment. *P<0.05, **P<0.01. All data are mean ± SEM.

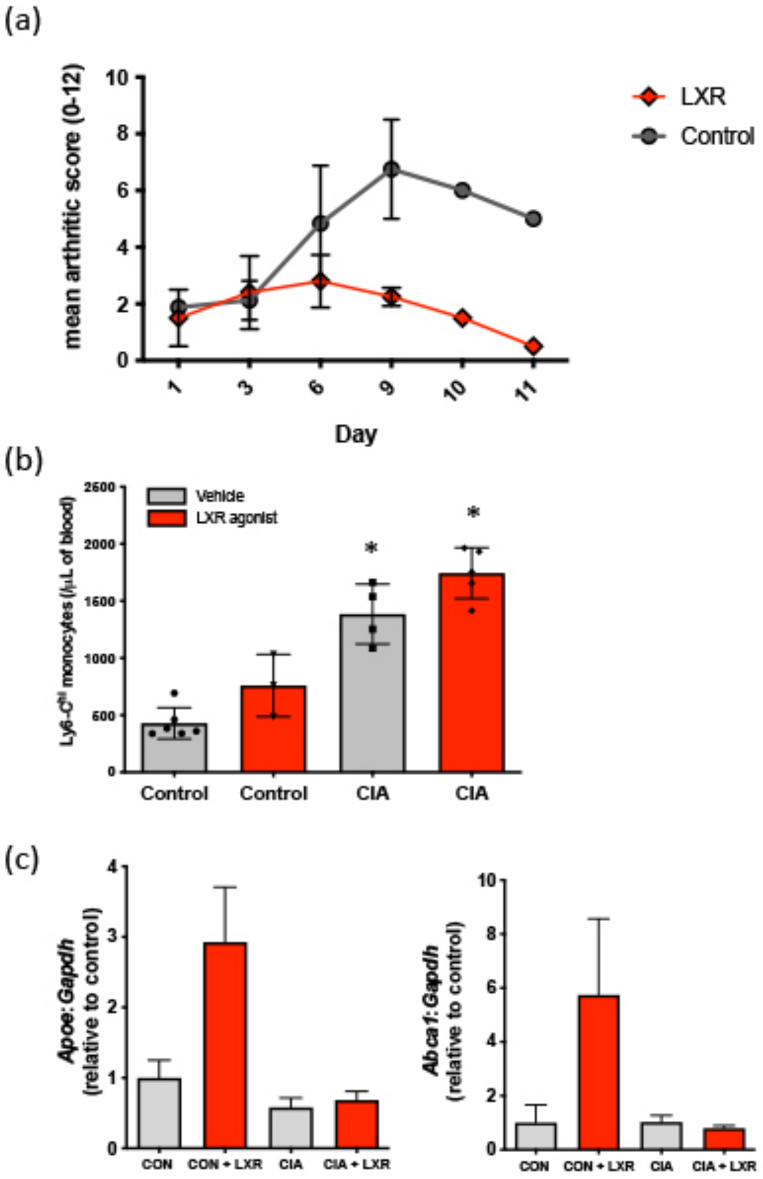

Supplementary Figure 2. WT mice with collagen-induced arthritis were administered vehicle or LXR agonist (TO901317, 25mg/kg, I.P., every 3 days) from day 2 of clinical symptoms. **(a)** Daily mean arthritic score, **(b)** Blood monocytes were quantified by flow cytometry and **(c)** mRNA expression from BM HSPCs. n = 3-5 mice/group, 1 cohort. All data are mean ± SEM. *P<0.05 vs respective control.

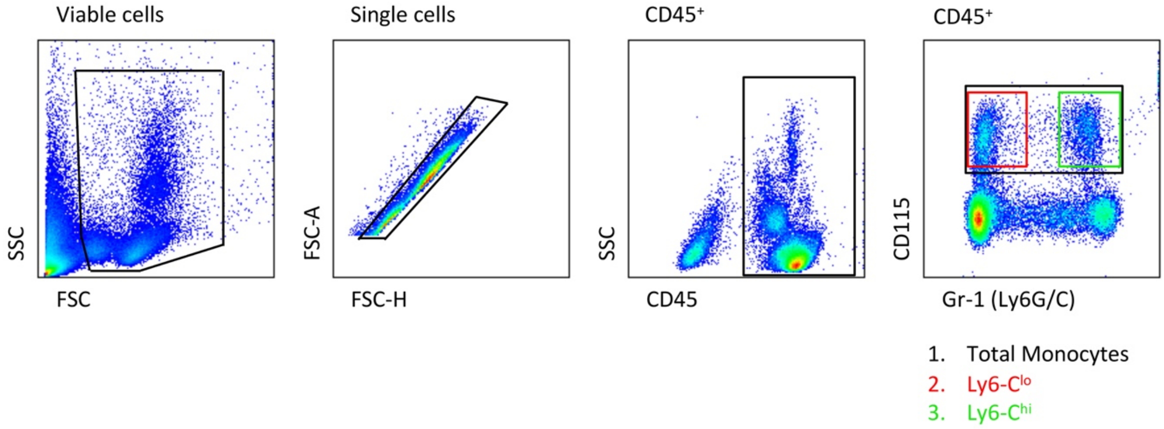

Supplementary Figure 3. Flow cytometric gating strategy to identify monocytes.

Supplementary table 1: Mouse gene primer sequences

Gene	Forward sequence	Reverse sequence
Gapdh	CGAAGGTGGAAGAGTGGGAG	TGAAGCAGGCATCTGAGGG
Apoe	GCCTAGCCGAGGGAGAGCCG	TGTGACTTGGGAGCTCTGCAGC
Abca1	CGTTTCCGGGAAGTGTCCTA	GCTAGAGATGACAAGGAGGATGGA
Abcg1	TTCCCCTGGAGATGAGTGTC	CAGTAGGCCACAGGGAACAT
Lxrα	GCTCTGCTCATTGCCATCAG	TGTTGCAGCCTCTCTACTTGGA
HmgcoaR	GGGAACTATTGCACCG	GTAGCCGCCTATGCTC
Ldlr	GAGGAACTG GCCGGCTGAA	GTG CTGGATGGGGAGGTCT
Srebf1	AATAAATCTGCTGTCTTGCG	CCTTCAGTGATTTGCTTTTG
Cyp27a	GGAGGATTGCAGAACTGGAG	TGCGGGACACAGTCTTTACTT
